# An Adult Mouse Model of *Vibrio cholerae*-induced Diarrhea for Studying Pathogenesis and Potential Therapy of Cholera

**DOI:** 10.1371/journal.pntd.0002293

**Published:** 2013-06-27

**Authors:** Sutthipong Sawasvirojwong, Potjanee Srimanote, Varanuj Chatsudthipong, Chatchai Muanprasat

**Affiliations:** 1 Department of Physiology, Faculty of Science, Mahidol University, Bangkok, Thailand; 2 Research Center of Transport Protein for Medical Innovation, Faculty of Science, Mahidol University, Bangkok, Thailand; 3 Graduate Study, Faculty of Allied Health Sciences, Thammasat University, Pathumtanee, Thailand; Institut Pasteur, France

## Abstract

Cholera is a diarrheal disease causing significant morbidity and mortality worldwide. This study aimed to establish an adult mouse model of *Vibrio cholerae*-induced diarrhea and to characterize its pathophysiology. Ligated ileal loops of adult mice were inoculated for 6, 9, 12 and 18 h with a classical O1 hypertoxigenic 569B strain of *V. cholerae* (10^7^ CFU/loop). Time-course studies demonstrated that the optimal period for inducing diarrhea was 12 h post-inoculation, when peak intestinal fluid accumulation (loop/weight ratio of ∼0.2 g/cm) occurred with the highest diarrhea success rate (90%). In addition, pathogenic numbers of *V. cholerae* (∼10^9^ CFU/g tissue) were recovered from ileal loops at all time points between 6–18 h post-inoculation with the diarrheagenic amount of cholera toxin being detected in the secreted intestinal fluid at 12 h post-inoculation. Interestingly, repeated intraperitoneal administration of CFTR_inh_-172 (20 µg every 6 h), an inhibitor of cystic fibrosis transmembrane conductance regulator (CFTR), completely abolished the *V. cholerae*-induced intestinal fluid secretion without affecting *V. cholerae* growth *in vivo*. As analyzed by *ex vivo* measurement of intestinal electrical resistance and *in vivo* assay of fluorescein thiocyanate (FITC)-dextran trans-intestinal flux, *V. cholerae* infection had no effect on intestinal paracellular permeability. Measurements of albumin in the diarrheal fluid suggested that vascular leakage did not contribute to the pathogenesis of diarrhea in this model. Furthermore, histological examination of *V. cholerae*-infected intestinal tissues illustrated edematous submucosa, congestion of small vessels and enhanced mucus secretion from goblet cells. This study established a new adult mouse model of *V. cholerae*-induced diarrhea, which could be useful for studying the pathogenesis of cholera diarrhea and for evaluating future therapeutics/cholera vaccines. In addition, our study confirmed the major role of CFTR in *V. cholerae*-induced intestinal fluid secretion.

## Author Summary

Cholera is a diarrheal disease causing significant morbidity and mortality worldwide. *Vibrio cholerae*, the causative agent of cholera, colonizes the intestine and induces massive intestinal fluid secretion through actions of its enterotoxins, especially cholera toxin. We have developed a ligated ileal loop model of *V. cholerae*-induced diarrhea in adult mice, in which inoculation of *V. cholerae* (10^7^ CFU/loop) consistently caused intestinal fluid secretion in 12 h. Interestingly, administration of CFTR_inh_-172, a small molecule CFTR inhibitor, completely abolished *V. cholerae*-induced intestinal fluid secretion, indicating that CFTR plays a pivotal role in the pathogenesis of diarrhea in this model. Furthermore, we demonstrated that intestinal fluid accumulation in this model was neither caused by increased intestinal paracellular permeability nor enhanced vascular leakage. This adult mouse model of *V. cholerae*-induced diarrhea could be very useful in the studies of pathogenesis of *V. cholerae* infection-induced diarrhea and in the evaluation of potential therapies/cholera vaccines.

## Introduction

Cholera is a life-threatening disease caused by intestinal infections with a gram-negative bacterium *Vibrio cholerae*. It has been estimated that cholera affects millions of people and causes several hundreds of thousands of death each year, especially in developing countries [1]. Importantly, the number of cholera cases has increased steadily in recent years, probably due to climate change and more frequent occurrence of natural disasters [2]. The main symptoms of cholera are profuse watery diarrhea and vomiting, which could lead to severe dehydration, hypovolemic shock and, with no appropriate treatment, death. The current treatment of cholera is the use of oral rehydration solution (ORS), with recommended use of antibiotics in moderate and severe cases [3]. However, these treatment strategies have some disadvantages. For instance, efficacy of ORS is reduced in children and elderlies, and even ineffective in severe cholera cases with excess intestinal fluid loss. Treatment benefit of antibiotics may be abated by the emergence of multidrug-resistant strains of *V. cholerae* [4]. Therefore, great efforts have been paid to develop novel adjunctive therapies of cholera, especially those that can reduce intestinal fluid loss by modulating intestinal fluid secretion and/or absorption processes [5], [6].

Diarrhea in cholera results from both direct and indirect (via enteric nervous system) effects of *V. cholera*e-derived enterotoxins, especially cholera toxin (CT), on intestinal epithelium [7]. Using cAMP as a second messenger, CT induces transepithelial Cl^−^ secretion, which in turn provides a driving force for Na^+^ and water secretion [8]. Both *in vitro* and *in vivo* experiments demonstrated that cystic fibrosis transmembrane conductance regulator (CFTR) mediates CT-induced apical Cl^−^ efflux into intestinal lumen and therefore represents a promising therapeutic target for treatment of cholera [9]. Interestingly, it was recently shown that, in addition to CFTR, unidentified inward rectifying Cl^−^ channels (IRC) might help mediate cAMP-activated Cl^−^ efflux across the apical membrane of intestinal epithelial cells [10]. To study the pathophysiology of diarrhea and to evaluate the potential anti-diarrheal therapies for cholera, most previous studies employed rodent and rabbit models of CT-induced intestinal fluid secretion [11]. However, extrapolation of the data obtained from these studies to explain the pathogenesis which would lead to therapeutic intervention of cholera in human proved difficult, since pathogenesis of cholera in human requires coordinated expression by *V. cholerae* of several other virulence factors other than CT [7]. Indeed, a number of investigations have recently suggested that other non-CT virulence factors produced by *V. cholerae* and inflammatory response induced by *V. cholerae* independently of CT may be involved in the pathogenesis of cholera [7], [12], [13].

To date, *V. cholerae* infection models have used both infant rabbits and infant mice [14]. Rabbit infants orally or intestinally inoculated with live *V. cholerae* have been shown to exhibit massive diarrhea and mucous secretion from goblet cells despite having intact microscopic architecture of intestinal epithelium, resembling pathology of cholera in human [15]. However, developing mouse models of cholera may be preferable to using rabbits for many reasons. Being small and with shorter lifespan, mice are relatively inexpensive, easy to handle and require smaller quantities of compounds/vaccines for therapeutic/preventive evaluation. Transgenic mice are also available for studying cholera pathophysiology. At present, the only established mouse model of *V. cholerae* infection is an infant mouse model of cholera induced by oral inoculation of *V. cholerae*. This infant mouse model has been used extensively for studying growth and colonization of *V. cholerae* in the intestine [16]. However, due to an absence of overt diarrhea and immature development of immune system, this model may not be suitable for studying pathogenesis of diarrhea or for evaluating anti-secretory therapeutics/vaccines of cholera [17]. With an attempt to establish adult animal models of cholera, Basu and Pickett [18] investigated fluid accumulation in ligated ileal loops (10-cm long) inoculated with live *V. cholera*e (Inaba strain 569B) or CT in various laboratory animals including gerbil, rat, hamster, guinea pig, chinchilla, cat and mouse. It was found that other types of animals developed profuse and consistent intestinal fluid secretion, whereas mice showed little or no fluid accumulation at 16 h after intestinal injection of bacteria or CT, despite normal replication of *V. cholerae* in mouse intestine. Based on the fact that *V. cholerae* do replicate in adult mouse intestine as shown by Basu and Pickett [18], and that CT has been found by a number of investigators including our groups to cause consistent and significant intestinal fluid secretion in closed 2–3 cm intestinal loops of adult mice [19], [20], we hypothesized that *V. cholerae* might induce diarrhea in adult mice under condition optimal for *V. cholerae* expression of their virulence factors, especially CT. In this study, we aimed to establish an adult mouse model of *V. cholerae*-induced diarrhea using a closed ileal loop approach, and to use it to investigate the underlying pathophysiology of diarrhea. A closed ileal loop approach was chosen because *V. cholerae* has been known to preferentially colonize distal small intestine particularly ileum [15] and secondly, a closed loop system allows direct and accurate quantitation of the intestinal fluid secretion, a pathophysiological hallmark of cholera. In this study, we demonstrated that *V. cholerae* (Inaba strain 569B) challenges into the ligated 2–3 cm ileal loops of adult ICR outbred mice consistently produced intestinal fluid accumulation in 12 h after inoculation in a CFTR-dependent manner.

## Methods

### Ethics statement

This study has been approved by the Institutional Animal Care and Use Committee of the Faculty of Science, Mahidol University (permit number 240). This study was performed in accordance with the recommendations in the Guide for the Care and Use of Laboratory Animals of the National Institutes of Health.

### Bacterial strains and chemicals

*Vibrio cholerae* 569B Inaba (classical, O1 strain) was used in this study. Bacteria were grown in Thiosulfate-Citrate-Bile-Sucrose (TCBS) agar and then duplicated using Luria Broth (LB) plate. To prepare the inoculum, *V. cholera* were cultured at 37°C overnight in LB. The overnight culture were then subcultured into fresh LB at the ratio of 1∶100 and grown to a mid-log phase. *V. cholerae* cells were collected by centrifugation and the pellet was re-suspended in phosphate buffered saline to obtain the inoculum containing 10^8^ CFU of *V. cholera*/ml. CT was purchased from List Biological Laboratories, Inc. (Campbell, CA, USA). CFTR_inh_-172 was purchased from Calbiochem (San Diego, CA, USA). Fluorescein isothiocyanate-linked dextran (FITC-dextran; molecular weight of 4.4 kDa) and other chemicals were obtained from Sigma-Aldrich (St. Louis, MO, USA).

### Establishment of ileal-ligated mouse model of cholera

Six-week-old ICR outbred mice (weight 30–35 g) were acquired from the National Laboratory Animal Center, Bangkok, Thailand. Before surgery, mice were fasted for 24 h and anesthetized by intraperitoneal injection of nembutal (60 mg/kg). While maintaining the body temperature at 37°C using a heating pad, a small abdominal incision was made and a loop of distal ileum was isolated by suture (2–3 cm in length). The closed ileal loop was instilled with 100 µl of phosphate buffered saline (PBS) or PBS containing *V. cholerae* (10^7^ CFU/loop). After abdominal closure by suture, mice were allowed to recover from anesthesia. At specific time points after bacterial inoculation (6, 12, 18, 24 h), the mice were re-anesthetized and ileal loops were exteriorized for measurements of weight/length ratio. Mice with intestinal weight/length ratio of al least 0.1 g/cm were considered as having positive diarrheal response. Diarrhea success rates (% diarrhea) were computed by dividing a number of surviving mice with intestinal loop weight/length ratio of at least 0.1 g/cm by a total number of surviving mice at a specific time point. In the study of CT-induced diarrhea, ileal loops were instilled with PBS or PBS containing CT (1 µg/loop), and loop weight/length ratio was quantified 4 h later. In some experiments, CFTR_inh_-172 (20 µg in 0.1 ml of DMSO) was intraperitoneally administered every 6 h until euthanasia. For bacterial colonization assays, ileal loops at various time points after *V. cholerae* inoculation were homogenized in PBS, followed by serial dilutions and plating on LB agar for colony forming unit (CFU) counting.

### Assay of cholera toxin in fecal fluid

Amount of CT in fecal fluid was quantified using GM_1_ enzyme-linked immunosorbent assay. Briefly, 100 µl of sample, standard CT in PBS or PBS alone was added to GM1-coated 96-well plates in duplicate and incubated at 37°C for 1 h. The plate was then washed three times with PBS before addition of goat-anti CT_b_ serum. An hour later, rabbit anti-goat globulin was added to the plate and incubated for 60 min. The substrate solution was then added and incubated for 30 min. The reaction was stopped by adding 50 µl of 3M NaOH and absorbance at 405 nm was measured using a microplate reader (BMG LABTECH, Victoria, Australia). The amount of CT was calculated according to the manufacturer's instructions.

### Electrophysiological analysis of mouse intestine

Ileal loops were opened longitudinally, washed with PBS and mounted in the Ussing chamber system (Physiologic Instruments Inc., San Diego, CA, USA.). Both apical and basolateral hemichambers were filled with Kreb's bicarbonate buffer containing (in mM) 120 NaCl, 25 NaHCO_3_, 3.3 KH_2_PO_4_, 0.8 K_2_HPO_4_, 0.5 MgCl_2_ and 10 glucose (pH 7.4). The solution was continuously bubbled with 5% CO_2_/95% O_2_ and maintained at 37°C. Electrical resistance of the intestinal tissues was calculated using values of passing current and transmembrane voltage recorded by a voltage/current clamp machine with Ag/AgCl electrodes and 1 M KCl agar bridges (Physiologic Instruments Inc., San Diego, CA, USA.).

### *In vivo* measurement of intestinal paracellular permeability

Intestinal paracellular permeability was estimated using the measurement of FITC-dextran (molecular weight 4.4 kDa) flux from the intestine to the blood as previously described [21]. Briefly, at 12 h after instillations of PBS, PBS containing *V. cholerae* (10^7^ CFU/loop) or PBS containing CT (1 µg/loop) into ileal loops, mice were anesthetized and intestinal fluid was removed and replaced with 0.2 mL of PBS containing 20 mg of FITC-dextran. Thirty min afterwards, mice were sacrificed and blood was collected by cardiac puncture. Blood was centrifuged at 3,000 g for 10 min (4°C) and the serum was collected for measurement of FITC-dextran using a fluorospectrophotometer (Bio-Tek Instrument, Helsinki, Finland). The amount of FITC-dextran in the sample was estimated from the standard curve generated by fluorometric measurements of FITC-dextran at known concentrations.

### Histological examination of mouse intestine

Intestinal tissues dissected from ileal loops were fixed for 24 h in 10% formaldehyde, followed by tissue dehydration using graded alcohol and paraffin embedding. The processed tissues were cut into 5 µm-thick sections and stained with hematoxylin and eosin (H&E) reagents or periodic-acid Schiff (PAS) reagent. Tissue slices were visualized under light microscope for histological analysis.

### Measurement of albumin in diarrheal fluid

Concentration of albumin in fluid in ileal loops was determined using a bromcresol purple (BCP) dye-binding method as previously described [22]. In brief, the obtained sample was mixed with the BCP reagent and the absorbance at 600 nm was measured using the automated Dimension RxL Max analyzer (Siemens Healthcare Diagnostics Inc., Tarrytown, NY, USA).

### Statistical analysis

The data were presented as mean ± standard error (S.E.). Statistical analyses for two-group and multiple comparisons were performed using Student's t test and one-way analysis of variance (ANOVA), followed by Bonferroni's post-hoc test, respectively. P value of <0.05 was considered statistically significant.

## Results

### Establishment of an adult mouse model of*V. cholerae*-induced diarrhea

**Figure 1:**
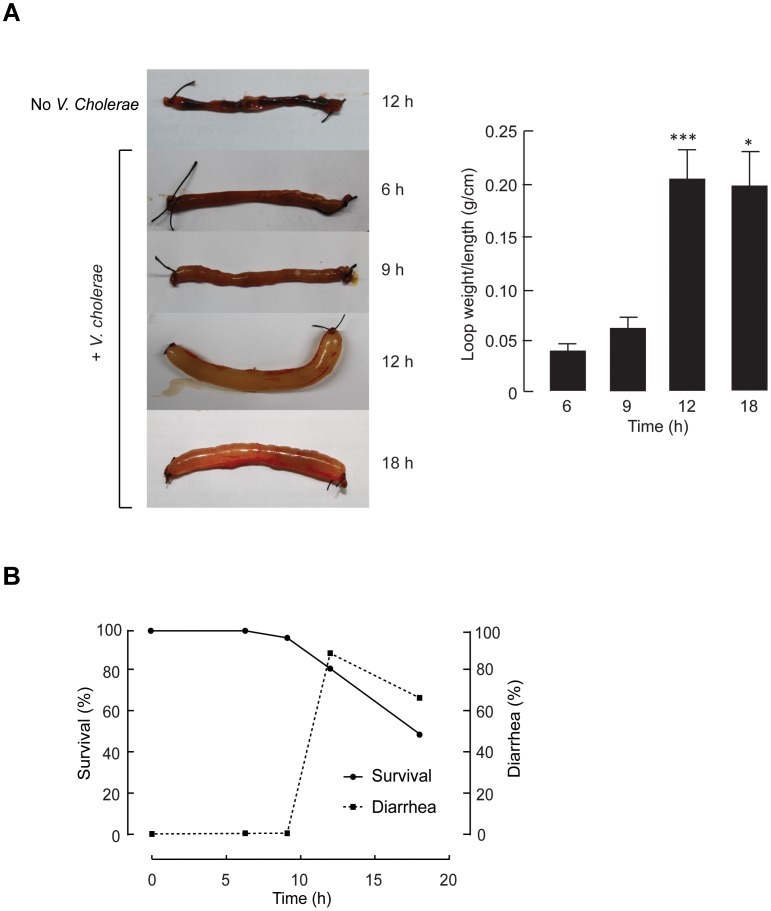
Characterization of the adult mouse model of cholera using ligated ileal loops inoculated with live*V.* *cholerae*. (A) Time course of intestinal fluid secretion induced by *V. cholerae* inoculation. 10^7^ CFU of *V. cholerae* were inoculated into ileal loops of anesthetized adult mice. At indicated time after bacterial challenge, loops were removed for measurements of loop weight/length ratio. *Left*, representative images of mouse ileal loops injected with saline or saline containing *V. cholerae* at specific time points post-inoculation. *Right*, summary of the data. Data were expressed as mean ± S.E. (n = 4–8 mice per group). *, P<0.05 and ***, P<0.01 compared with 6 h. (B) Survival and diarrhea success rates of the *V. cholerae*-inoculated mice over the indicated periods of time after inoculation (n = 39 mice).

*Vibrio cholerae* 569B of classical O1 Inaba strains (10^7^ CFU/loop), a bacterial strain known to possess high diarrheagenic potential [18], was inoculated into the ligated ileal loop of 2–3 cm length. This length of intestinal loops was previously shown by our group to be optimal for detecting CT-induced excessive intestinal fluid secretion in adult mice [19], [20]. We first sought to determine the optimal incubation periods for *V. cholerae*-induced intestinal fluid secretion by analyzing three parameters, i.e., the amount of intestinal fluid secretion, percent of mice developing fluid accumulation (loop weight/length ratio of at least 0.1 g/cm), and survival rate of mice at different time points (6, 9, 12 to 18 h) after *V. cholerae* inoculation. As depicted in Fig. 1A, intestinal fluid secretion increased over time and reached its peak at 12 h after *V. cholerae* inoculation (loop weight/length ratio = 0.203±0.037 g/cm). This level of intestinal fluid secretion was sustained until 18 h post-inoculation (loop weight/length ratio = 0.192±0.042 g/cm). Diarrhea success rate, which indicated model reproducibility, and survival rate of mice were 90% and 80% at 12 h post-inoculation, and 68% and 50% at 18 h post-inoculation, respectively (Fig. 1B). Therefore, an optimal incubation period for induction of diarrhea by *V. cholerae* was 12 h. It was also noted that higher inoculation doses of *V. cholerae* (10^8^–10^9^ CFU/loop) caused lethality in more than 80% of mice within 6 h after bacterial challenge without inducing fluid secretion. We thought that circulatory failure resulting from *V. cholerae*-induced systemic inflammatory response might account for this death. Subsequent experiments were performed to characterize the pathophysiology of this cholera model.

### *V. cholerae* colonization and cholera toxin (CT) expression in mouse intestine

**Figure 2:**
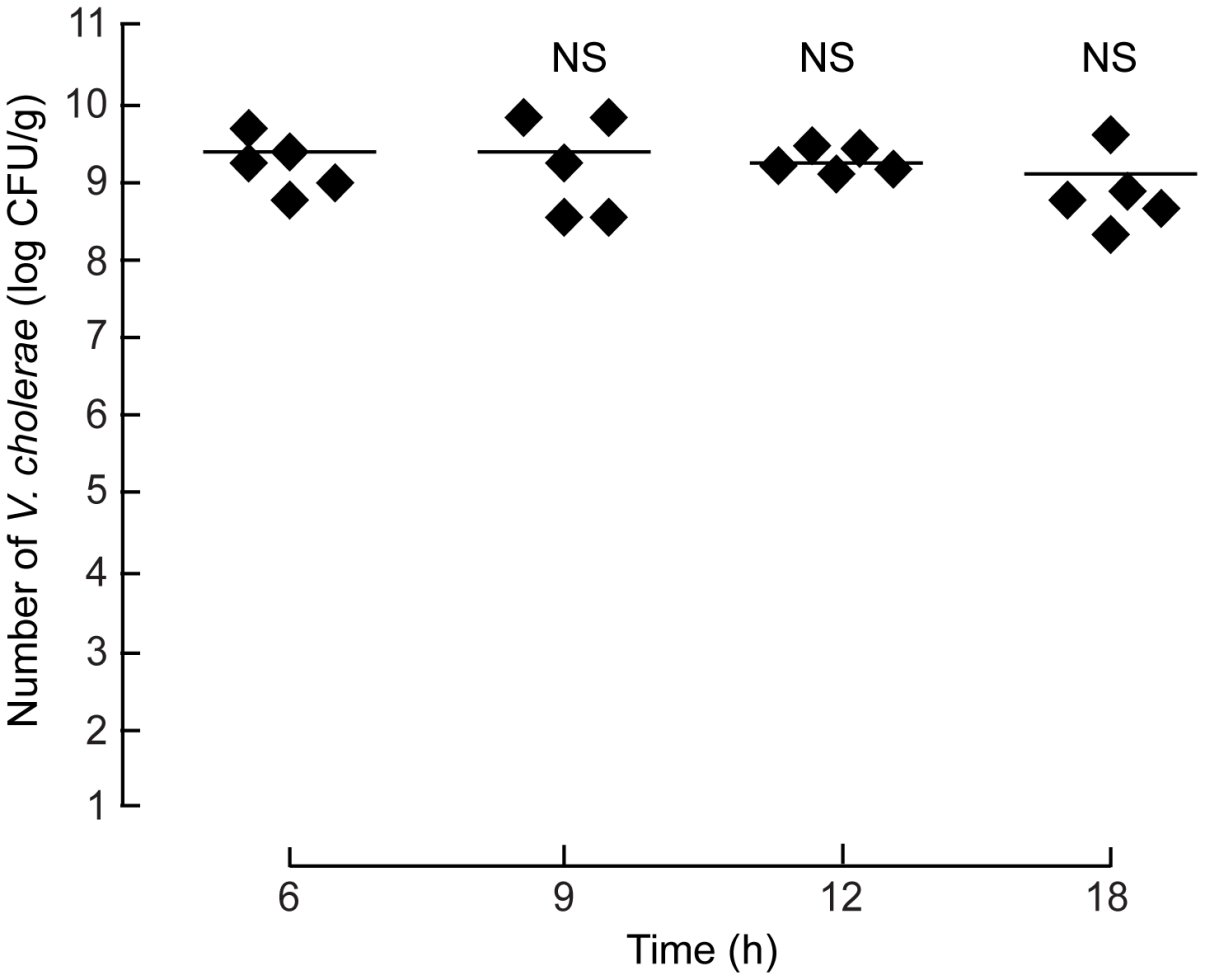
Quantitation of*V.* *cholerae* in mouse ileal loops. Ileal loops were inoculated with 10^7^ CFU of *V. cholerae*. Amount of *V. cholerae* recovered from the homogenates of ileal loops was measured at indicated time after *V. cholerae* inoculation (n = 5 mice per group). Solid lines represent mean values of the number of recovered bacteria. NS, non-statistical difference compared with 6 h.

Pathogenesis of diarrhea in cholera required *V. cholerae* colonization and production of CT [14]. To gain insight into the mechanism of diarrhea in this model, the amount of *V. cholerae* in the intestinal loops (intestinal fluid plus intestinal tissues) and CT in intestinal fluid were determined. As shown in Fig. 2, the number of *V. cholerae* recovered from the intestinal loops at all time points (6 h, 9 h, 12 h and 18 h) after inoculation was ∼10^9^ CFU/gram of tissues. This amount of *V. cholerae* was comparable to that recovered from the intestine of infant rabbit models of *V. cholerae* infection-induced diarrhea [15], suggesting that *V. cholerae* was capable of colonizing the intestine in our mouse cholera model. Using GM_1_ ELISA assays, the amount of CT in intestinal fluid was found to be 1.688±0.563 µg/ml at 12 hour post-inoculation, whereas none was detected in the ileal loop instilled with PBS (no *V. cholerae*). These data indicated that *V. cholerae* established colonization and expressed CT in the closed ileal loop in this mouse cholera model.

### Pathophysiology of*V. cholerae*-induced diarrhea

**Figure 3:**
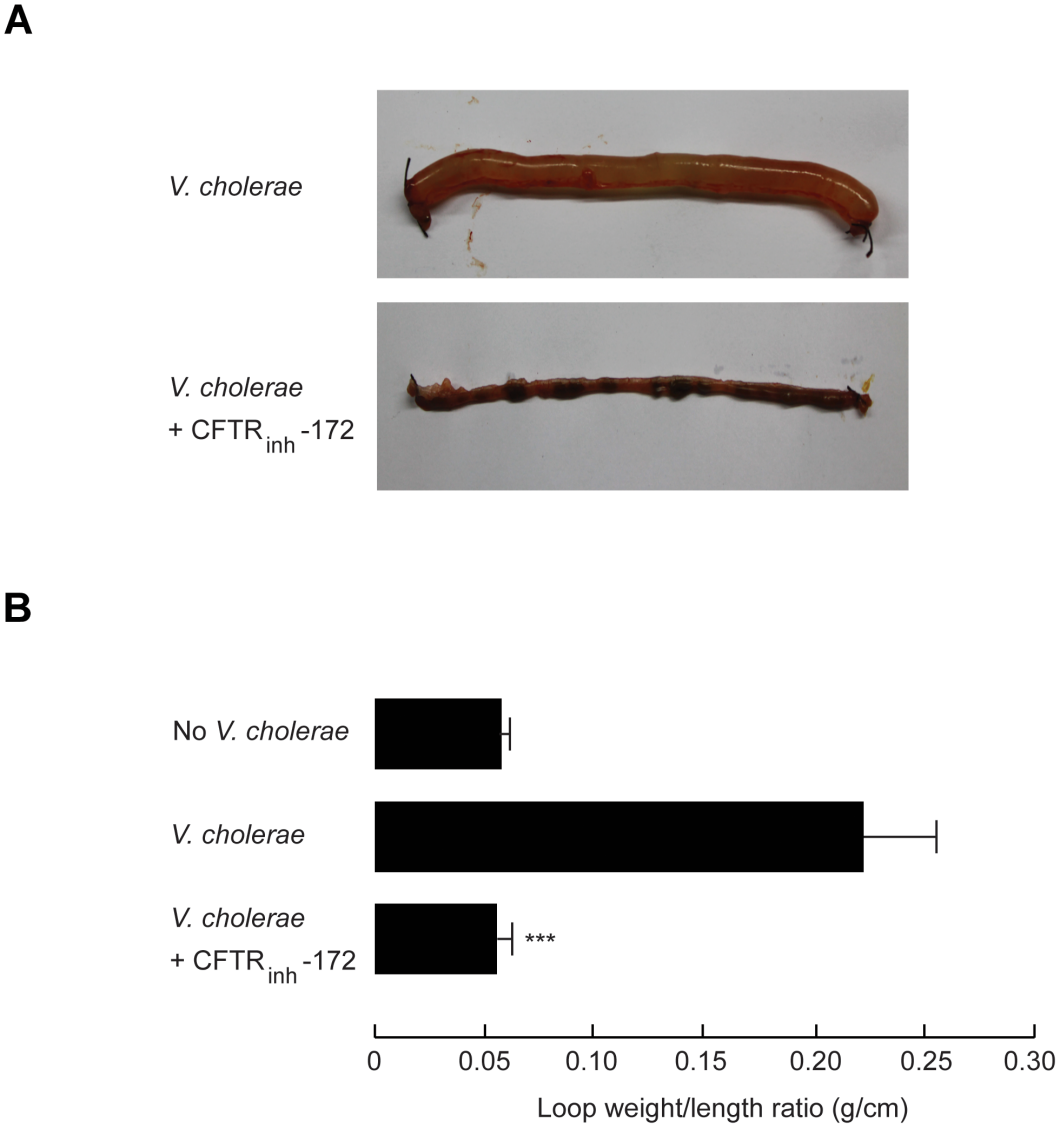
Effect of CFTR_inh_-172 on *V.* *cholerae*-induced intestinal fluid secretion in adult mice. After inoculation of *V. cholerae* into ileal loops, 20 µg of CFTR_inh_-172 was administered into mouse peritoneal cavity every 6 h. At 12 h after *V. cholerae* challenge, ileal loops were removed for measurement of loop weight/length ratio. (A) Representative photographs of ileal loops after indicated treatments. (B) Loop weight/length ratio with indicated treatments. Data were expressed as mean ± S.E. (n = 4). ***, P<0.01 compared with *V. cholerae*-inoculated control.

It has been known that CFTR-mediated transepithelial Cl^−^ secretion provides the driving force for CT-induced intestinal fluid secretion [9]. To estimate the relative contribution of CFTR-mediated Cl^−^ secretion to the pathogenesis of diarrhea in this model, the effect of CFTR_inh_-172, a CFTR inhibitor, on *V. cholerae*-induced intestinal fluid secretion was investigated. In this experiment, CFTR_inh_-172 (20 µg) was intraperitoneally administered at the time of *V. cholerae* inoculation and 6 h thereafter, and mice were sacrificed for measurements of intestinal fluid secretion (loop weigh/length ratio) at 12 h after *V. cholerae* inoculation. This dosage regimen of CFTR_inh_-172 was used because it was previously shown to produce maximal degrees of inhibition of CT-induced intestinal fluid secretion in mice (∼90% inhibition) [23]. Interestingly, we found that treatment with CFTR_inh_-172 completely prevented *V. cholerae*-induced intestinal fluid accumulation (Fig. 3). In addition, effects of CFTR_inh_-172 on *V. cholerae* growth were determined both *in vitro* and *in vivo*. Addition of *V. cholerae* for 24 h with CFTR_inh_-172 (at 20 µM, a concentration at which CFTR_inh_-172 is maximally soluble in saline and fully inhibits CFTR-mediated Cl^−^ secretion in intact intestinal epithelia) to the 5×10^8^ CFU/ml of *V. cholerae* in Mueller Hinton Broth did not affect *V. cholerae* growth as enumerated by a spread plate method (data not shown). Moreover, amounts of *V. cholerae* recovered from *V. cholerae*-inoculated ileal loops of CFTR_inh_-172-treated mice were not statistically different from that of control (*V. cholerae* inoculation with no CFTR_inh_-172 treatment) (data not shown). These data indicated that CFTR_inh_-172 inhibited *V. cholerae*-induced intestinal fluid secretion without affecting *V. cholerae* growth in ileal loops *in vivo*.

**Figure 4:**
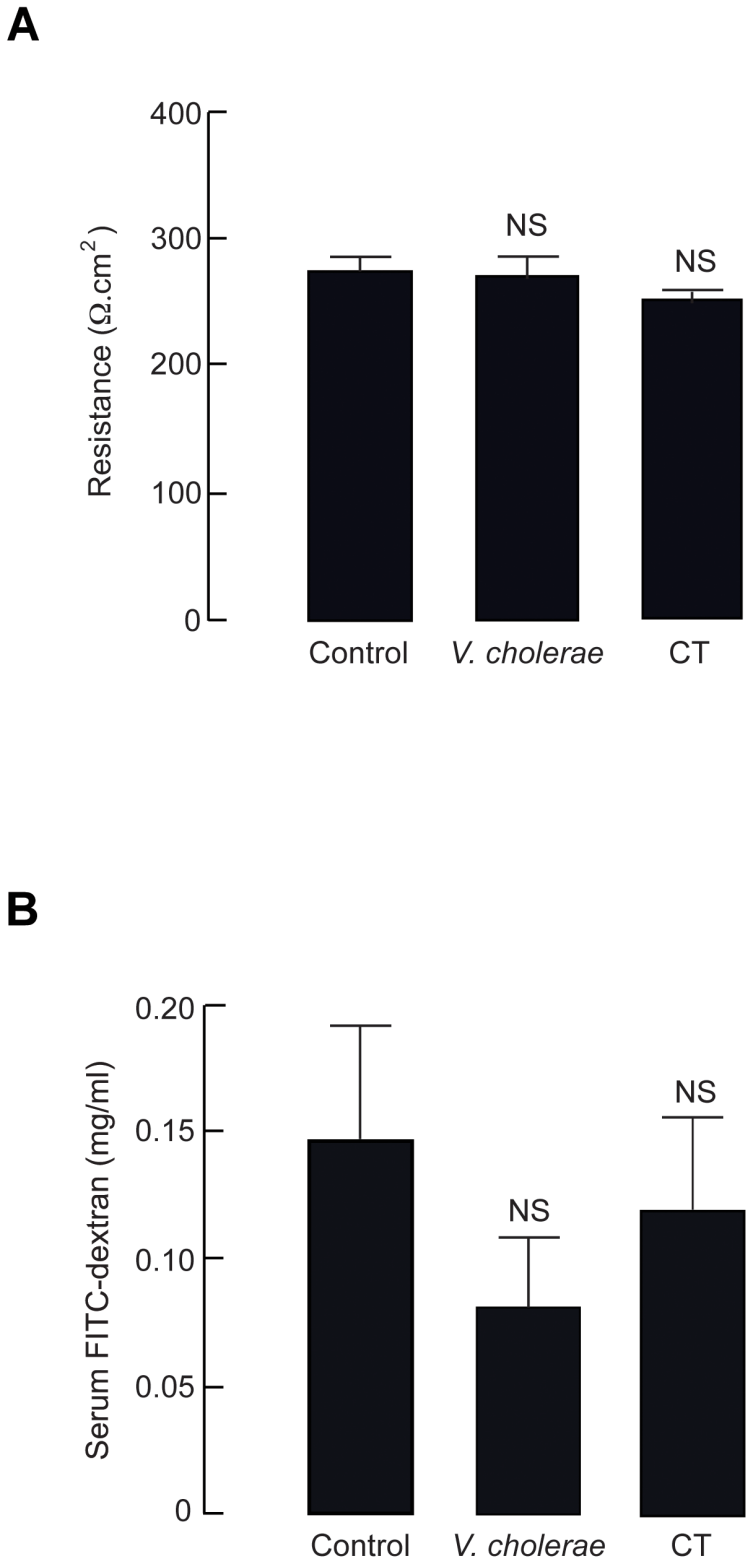
Analysis of intestinal paracellular permeability in the established model. (A) Transepithelial electrical resistance (TEER) of intestinal tissues. At 12 h after instillation of saline (control), *V. cholerae* (10^7^ CFU/loop), or cholera toxin (CT, 1 µg/loop) into ileal loops, intestinal tissues were measured for TEER using Ussing chamber systems. Data were expressed as mean ± S.E. (n = 5 mice per group) NS, non-statistical difference compared with control. (B) *In vivo* measurements of intestinal paracellular permeability using FITC-dextran assays. At 12 h after instillation of saline (control), *V. cholerae* (10^7^ CFU/loop) or cholera toxin (CT, 1 µg/loop) into ileal loops, intestinal fluid was removed and replaced with FITC-dextran (molecular weight of 4.4 kDa). Thirty min thereafter, blood was taken by cardiac puncture for analysis of serum FITC-dextran level. Data were expressed as mean ± S.E. (n = 4–8 mice per group). NS, non-statistical difference compared with control.

Possibility of paracellular leakage as a cause of *V. cholerae*-induced intestinal fluid accumulation was investigated using both *ex vivo* measurements of transepithelial electrical resistance (TEER) of mouse intestine by Ussing chamber systems and *in vivo* FITC-dextran trans-intestinal flux assays. As shown in Fig. 4A, TEER of *V. cholerae*-inoculated intestinal tissues was not significantly different from that of saline-injected control and CT-injected groups (268.87±13.80 Ω·cm^2^, 266.21±19.59 Ω·cm^2^ and 240.79±4.20 Ω·cm^2^, respectively). In order to confirm that *V. cholerae* infection had no effect on the intestinal paracellular permeability, *in vivo* FITC-dextran trans-intestinal flux assays were performed. In this experiment, FITC-dextran was injected into the intestinal loops and serum level of FITC-dextran was measured 30 min thereafter. It was found that *V. cholerae* infection had no effect on serum FITC-dextran compared with saline control and CT-injected groups (Fig. 4B). Taken together, our results suggested that paracellular permeability of the mouse intestine was unaltered by *V. cholerae* infection and CT exposure.

To determine the contribution of vascular leakage to *V. cholerae*-induced intestinal fluid accumulation in this model, the amount of the plasma protein albumin was determined in fecal fluids of both control (no *V. cholerae*) and *V. cholerae*-inoculated mice. It was found that levels of albumin in fecal fluid of both control and *V. cholerae*-inoculated intestinal loops were under detection limit (<0.3 g/dL). This result indicated that vascular leakage did not contribute to the development of diarrhea in this experimental model of cholera.

### Histological analysis of intestinal tissues after*V. cholerae* infection

**Figure 5:**
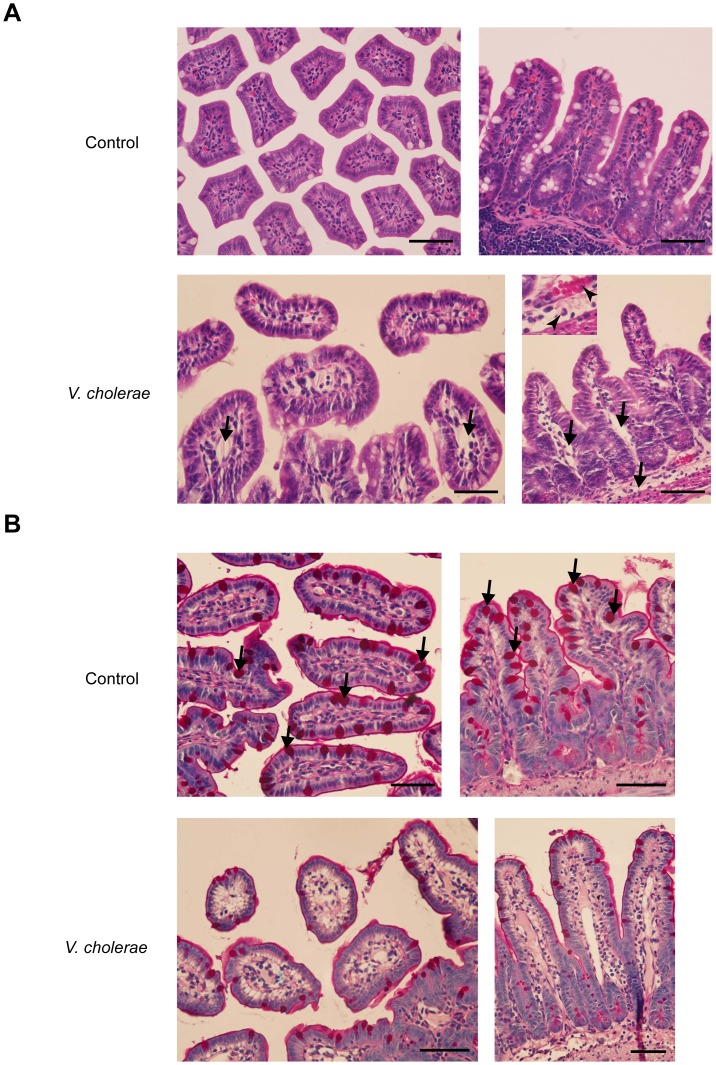
Histological analysis of intestinal sections. At 12 h after administration of saline (control) or inoculation with *V. cholerae* (10^7^ CFU/loop) into ileal loops, ileal loops were removed for histological examination. (A) Representative images of hematoxylin & eosin stained tissues. Arrows indicate edematous submucosa. Arrowheads indicate inflammatory cell infiltration. Photographs were taken at 40× magnification. Scale bar = 500 µm. (n = 5 mice per group) (B) Representative photographs of periodic-acid Schiff (PAS)-stained ileal tissues. Arrows indicate mucin-containing goblet cells, which were positively stained with magenta color. Photographs were taken at 40× magnification. Scale bar = 500 µm. (n = 5 mice per group).

To investigate the microscopic changes of the intestinal tissues after infection with *V. cholerae*, the *V. cholerae*-infected and normal saline-injected control loops were subjected to histological examination. As shown in Fig. 5A, *V. cholerae*-infected tissues showed diffuse edema of the submucosal area and marked enlargement of the internal structures of intestinal villi compared to control. Furthermore, vascular congestion (arrows, Fig. 5A) and inflammatory cell infiltration (arrowheads, Fig. 5A) were noted in *V. cholerae*-infected intestinal tissues. In addition, mucin secretion, a phenotypic response of mucin-containing goblet cells to *V. cholerae* infection, was analyzed by periodic acid-Schiff (PAS) staining, which detects mucin in tissues. As depicted in Fig. 5B, *V. cholerae*-infected intestine showed a marked reduction in PAS-positive goblet cells (arrows) compared with control, indicating empty goblet cells after secreting mucin in response to *V. cholerae* infection. These findings indicated that this animal model resembles human cholera in a histological context.

## Discussion

To date, several animal models have been developed for cholera research. Most of these models are generated using both live *V. cholerae* or its virulence factors especially CT. The only *V. cholerae* infection model that manifests severe watery diarrhea was the infant rabbit model [15]. In this study, we established a ligated ileal loop model of *V. cholerae* infection in adult mice. In this model, inoculation of 10^7^ CFU of *V. cholerae* into closed ileal loops of 2–3 cm length led to massive intestinal fluid secretion, with the optimal period for induction of fluid secretion being 12 h after inoculation. Importantly, CT at the amount reported to elicit intestinal fluid secretion was detected in the intestinal fluid. Furthermore, we provided the evidence that this model might be beneficial for studying pathophysiology of fluid secretion during *V. cholerae* infection and for evaluation of anti-secretory therapy of cholera.

More than four decades ago, it was reported that *V. cholerae* inoculation (5×10^7^ CFU/loop) into 10 cm-long ileal loops failed to cause fluid secretion [18]. Using the same strains of mice and *V. cholerae*, but shorter ileal loops, we successfully established a reproducible *V. cholerae*-induced diarrhea model in adult mice. Successful establishment of diarrhea model in the present study may be due to 1) optimal proportion of the amount of inoculated *V. cholerae* and length (i.e. volume) of ileal loops, and 2) optimal environment in distal ileum. Proportion between numbers of *V. cholerae* and volume of ileal loops determines the density of *V. cholerae*, which in turn affects *V. cholerae* expression of virulence factors including a colonizing factor toxin-coregulated pilus (TCP) and CT via quorum sensing mechanisms [24]. In addition, the shorter ileal loop mostly composed of distal ileum may create optimal environment for *V. cholerae* expression of virulence factors. These environmental factors include concentrations of bile and HCO_3_^−^ in the ileal loops. *V. cholerae* expression of TCP and CT has previously been shown to be repressed and enhanced by unsaturated fatty acids in bile (e.g. arachidonic, linoleic, and oleic acids) and HCO_3_^−^, respectively [25], [26]. The ability of *V. cholerae* to multiply and express CT in ileal loops in our study suggests the existence of environmental signals suitable for virulence expression of *V. cholerae*.

Time course of intestinal fluid secretion in the established cholera model is in accord with the prior knowledge regarding pathogenesis of *V. cholerae*-induced diarrhea. In this study, we found that 12-h period of *V. cholerae* incubation was required to obtain maximal intestinal fluid secretion. Since it was previously reported that maximal fluid secretion of mouse ileum required at least 6 h of exposure to CT [23], it is therefore estimated that *V. cholerae* colonized and expressed CT in the ileal loops within 6 h after inoculation in our model of study. In support of this notion, previous study demonstrated that *V. cholerae* expression of virulence factors including colonizing factors (e.g. TCP) and CT occurred within 4 h after bacterial inoculation [27]. Furthermore, attainment of the pathogenic amount of *V. cholerae* (∼10^9^ CFU/g tissue) at 6 h post-inoculation in our study affirmed that *V. cholerae* colonization and virulence expression had occurred within the first 6-h of *V. cholerae* exposure. Interestingly, time course of diarrhea in our model was similar to that in canine cholera model, in which diarrhea developed at 6–12 h after inoculation of *V. cholerae* into duodenal lumen [28].

Investigation of pathophysiological mechanisms underlying diarrhea in this new cholera model has highlighted CFTR as a therapeutic target for treatment of cholera. A number of previous investigations have demonstrated that CFTR inhibitors reversed CT-induced intestinal fluid secretion in both rats and mice [29]. However, the exact therapeutic value of CFTR inhibitor in the treatment of cholera has been elusive, since anti-diarrheal efficacy of CFTR inhibitors has never been investigated in animal models of *V. cholerae*-induced diarrhea. In fact, pathophysiology of diarrhea induced by CT and *V. cholerae* might be different, as *V. cholerae* can express several enterotoxins other than CT, which may participate in development of profuse diarrhea in cholera [7]. In support of this statement, some experimental therapeutics that were effective in mouse closed loop models of CT-induced fluid secretion such as racecadotril, an enkephalinase inhibitor, was subsequently found to produce no benefit in cholera patients, indicating the need to evaluate potential utility of anti-diarrheal therapeutics using *V. cholerae*-induced diarrhea models [30]. Of particular importance, a recent study in human intestinal epithelial (T84) cells revealed the signaling crosstalk between cAMP and Ca^2+^, which thus raised the possibilities that calcium-activated Cl^−^ channel (CaCC)-mediated transepithelial Cl^−^ secretion may contribute to fluid secretion in cholera [10]. In this study, we showed that CFTR_inh_-172, a small molecule CFTR inhibitor which inhibits CFTR but not CaCC [31], completely abrogated *V. cholerae*-induced intestinal fluid secretion. This finding indicates the predominant role of CFTR-mediated Cl^−^ secretion in providing the driving force for intestinal fluid secretion during *V. cholerae* infection. In addition, we provided evidence that paracellular and vascular leakage were not involved in the pathogenesis of *V. cholerae*-induced intestinal fluid secretion in this cholera model. This observation, together with the inhibitory effect of CFTR_inh_-172 on *V. cholerae*-induced intestinal fluid secretion, suggests that CFTR-mediated Cl^−^ secretion is the major pathophysiological event leading to secretory diarrhea at least in the early phase (∼12 h) of *V. cholerae* infection and could be the important target of anti-secretory drug for cholera. In addition, this diarrhea model might be beneficial for cholera research, especially for further investigation of the underlying pathophysiology of secretory diarrhea in cholera, and for evaluation of anti-secretory therapy of cholera. Importantly, due to the maturation of immune system in the adult mice, this cholera model could be used for evaluating preventive efficacy of cholera vaccine

In summary, we have established the ligated ileal loop model of *V. cholerae*-induced diarrhea in adult mice. Pathogenesis of diarrhea in this cholera model results from CFTR-mediated transepithelial Cl^−^ secretion with no involvement of disrupted intestinal barrier function or vascular leakage. This animal model may be beneficial for studying pathogenesis of diarrhea and evaluating potential anti-secretory therapies as well as vaccines for cholera.
